# Milestones in *Bacillus subtilis* sporulation research

**DOI:** 10.15698/mic2021.01.739

**Published:** 2020-11-27

**Authors:** Eammon P. Riley, Corinna Schwarz, Alan I. Derman, Javier Lopez-Garrido

**Affiliations:** 1 Division of Biological Sciences, University of California, San Diego, La Jolla, CA, USA.; 2 Max Planck Institute for Evolutionary Biology, Plön, Germany.

**Keywords:** Bacillus subtilis, sporulation, genetics, SpoIIIE, SpoIIIA, SpoIIQ, sporulation history

## Abstract

Endospore formation has been a rich field of research for more than a century, and has benefited from the powerful genetic tools available in *Bacillus subtilis*. In this review, we highlight foundational discoveries that shaped the sporulation field, from its origins to the present day, tracing a chronology that spans more than one hundred eighty years. We detail how cell-specific gene expression has been harnessed to investigate the existence and function of intercellular proteinaceous channels in sporulating cells, and we illustrate the rapid progress in our understanding of the cell biology of sporulation in recent years using the process of chromosome translocation as a storyline. Finally, we sketch general aspects of sporulation that remain largely unexplored, and that we envision will be fruitful areas of future research.

## INTRODUCTION

Since their discovery, bacterial endospores (a.k.a. spores) have attracted the attention of the scientific community and, at times, even the general public. This should not come as a surprise, as some properties of endospores could well be in the headlines of the popular press. Bacterial endospores are among the most resilient cell-types known, and can survive for very long periods of time without any nutrients. In fact, it has been claimed that viable endospores were isolated from the gut of extinct bees buried in Dominican amber more than 25 million years ago [1], evoking the imagery of Jurassic Park [2]. Furthermore, the use of *Bacillus anthracis* (anthrax) spores in biological warfare and, more recently, in bioterrorist attacks has brought the bacterial spore to the attention of modern society [3]. The study of endospore formation, however, is full of fascinating discoveries that, while subtle enough to escape the attention of the mainstream media, have contributed to shape our current perception of bacterial cells. This review aims to discuss some of the key findings that have shaped the sporulation field, through the lens of bacterial genetics.

## A BRIEF HISTORY OF ENDOSPORE FORMATION

**Figure 1:**
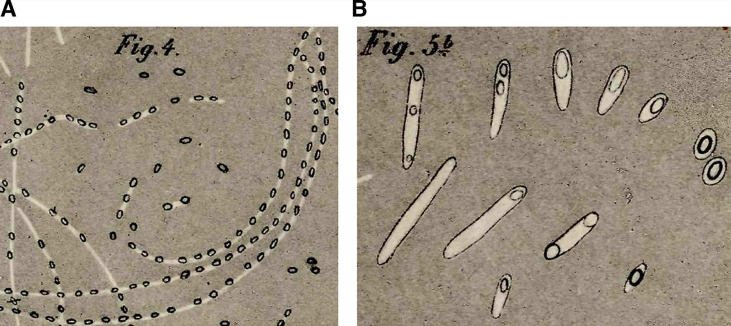
FIGURE 1: Early depictions of sporulation and germination in *Bacillus spp.* In one of his most well-known studies [6], Robert Koch investigated the etiology of anthrax. **(A)** Koch followed the sporulation process in *B. anthracis*. He placed a slice of spleen containing the *Bacillus* into cow sera or aqueous humor, and incubated the specimens at 35-37°C in an incubator that he himself had constructed. During the incubation, he observed the cells growing into string-like structures. After 10-15 h, these strings contained light refracting bodies, which he identified as spores. Koch depicted the structures in this sketch and likened them to fragile strings of pearls. The strings gradually decomposed, the spores were released and sank to the bottom of the droplet where they accumulated and could be kept for weeks. **(B)** Koch followed spore germination and outgrowth, and determined that spores can form viable cells. He fixed dried spores on a slide and incubated the sample with aqueous humor. After 3-4 hours, he observed various stages of germination and outgrowth, which are depicted in Cohn's sketch. Koch described spores as egg-shaped structures, enclosed by a thin layer of protoplasm that he called bright matter. He hypothesized that during outgrowth this bright matter stretched and became the vegetative cell, whereas the spore remained at one cell pole, lightened, and shrank, before it dissociated and disappeared. He proposed that the spore core consists of an oily substance necessary for the cell to resume vegetative growth.

Arguably the most important milestone in endospore research was the actual discovery of endospores. The first reported observation of bacterial endospores dates back to 1838, when Christian Gottfried Ehrenberg noted refractile bodies inside bacterial cells [4]. It was not until nearly four decades later that endospores started to be characterized in seminal studies by Ferdinand Cohn and Robert Koch. Cohn initiated the study of the resistance properties of spores with the observation that *B. subtilis* spores survive periods of boiling [5]. Koch, in collaboration with Cohn, followed the sporulation-germination cycle in *B. anthracis* [6], and realized that spores and vegetative cells are different cellular forms of the same bacterial species, and that cells can interconvert between these two forms, from vegetative cells to spores via sporulation, and from spores to vegetative cells via germination. Their hand-drawn sketches of these processes are shown in **Fig. 1**.

At the same time, John Tyndall independently postulated the existence of latent bacterial forms able to survive extreme temperatures. He described a sterilization technique (now designated “tyndallization”) that eradicates spores from culture media through the application of intermittent heating periods that kill vegetative cells, interspersed with periods of no heat that permit the spores that survive to germinate into vegetative cells, which are then killed during subsequent heating periods [7].

Throughout the next few decades, researchers attempted to characterize the process of endospore formation in different bacterial species, but it was difficult for them to achieve a unified understanding due to the limitations of the optical microscopy and staining methods available at that time [8, 9]. Some researchers, led by Koch, thought that spores were formed through the growth of a single refractile granule inside the cytoplasm of the vegetative cell [6]. A related model postulated that spores were not the result of the growth of a single granule, but of the fusion of multiple sporogenic granules inside the vegetative cell [10]. These putative sporogenic granules were probably cytoplasmic inclusion bodies that are present in various species of endospore forming bacteria, and are unrelated to spore formation. A different model, which comports better with our current perception of endospore formation (see below), proposed that spores were formed through the condensation of part of the vegetative cell protoplasm, which gained refractility over time [11]. A shared feature of these models, however, was that spore formation happened close to a single cell pole.

The advent of electron microscopy allowed for a more comprehensive cytological model for sporulation to be developed. In the late 1950's and early 60's, Young and Fitz-James provided detailed cytological descriptions of endospore formation in *Bacillus cereus* [12, 13]. Electron microscopy studies on sporulation in other *Bacillus* and *Clostridium* species were published shortly thereafter [14–16]. By the mid 60's, the field had achieved a consensus view of the cytological transformations leading to endospore formation, which are highly conserved among different species.

**Figure 2:**
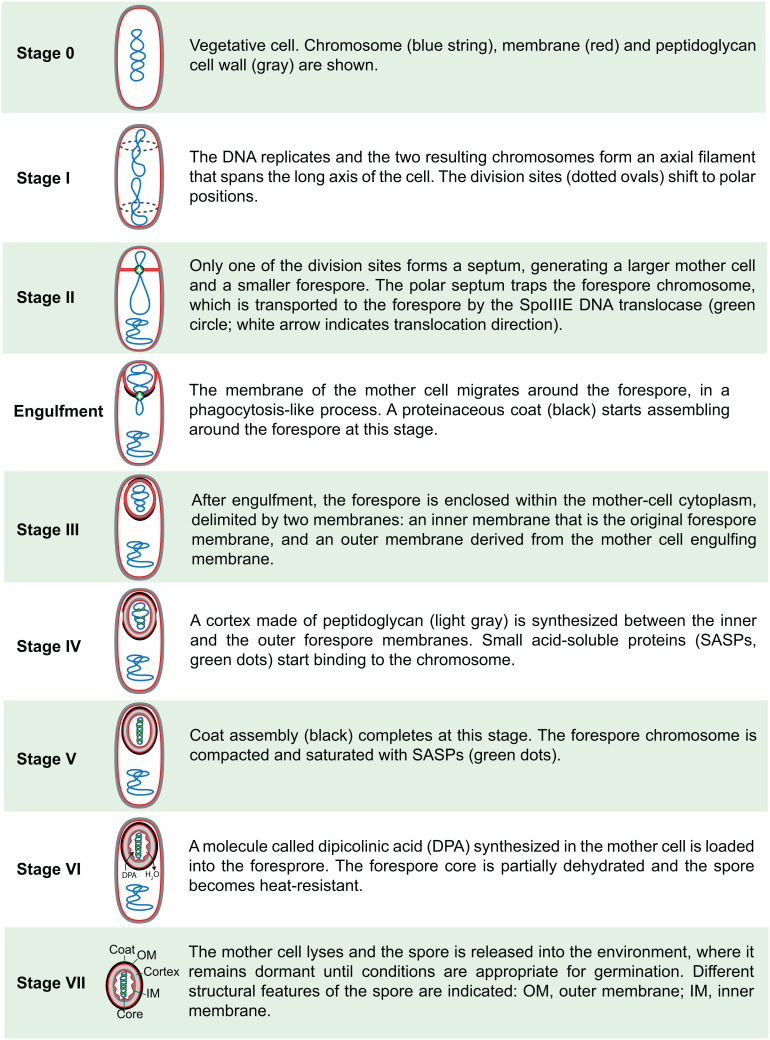
FIGURE 2: Sporulation stages in *B. subtilis*.

In 1965, Antoinette Ryter defined six different stages of sporulation [17], based on her own studies of *B. subtilis* and on those of Young and Fitz-James in *B. cereus*, and this classification was later expanded to eight stages [18, 19]. An up-to-date description of the different stages in *B. subtilis*, including details derived from fluorescence microscopy and genetic studies, is shown in **Fig. 2**. It is important to note that this classification represents discrete cytological steps of a process that is actually continuous. Thus, not only do several steps occur concomitantly, but also details about the transition between different stages are often neglected. For example, the transition between stages II and III happens through a phagocytosis-like process called engulfment, in which the membrane of the mother cell migrates around the forespore, generating the cell within a cell that is a hallmark of endospore formation (**Fig. 2**); the synthesis of the different spore layers is also gradual and starts shortly after polar septation (**Fig. 2**) [20]. Nevertheless, the classification proposed by Ryter represents a conceptual framework that facilitated the subsequent genetic dissection of spore formation.

## *B. SUBTILIS* AND THE GENETIC ANALYSIS OF SPORULATION MUTANTS

During the first half of the 20^th^ century, sporulation was studied simultaneously in different bacterial species. Two major discoveries, however, positioned *B. subtilis* to be the ideal model system for characterizing the process of sporulation during the second half of the century: in 1958 John Spizizen reported that *B. subtilis* can be transformed with DNA [21]; shortly thereafter, Thorne and Takahashi independently isolated phages capable of mediating generalized transduction in the same organism [22–25]. These powerful tools opened new avenues for genetic studies, and several laboratories turned their attention to *B. subtilis* to perform thorough genetic analyses of sporulation.

Mutants unable to form endospores (asporogenous), or with significantly reduced ability to form spores (oligosporogenous) have been reported for different species of endospore-forming bacteria ever since the inception of sporulation research [26]. The defining characteristic of oligosporogenous mutants is that the spores produced by them yield vegetative cells with equally low ability to produce spores. In contrast, asporogenous mutants, by definition, do not form viable spores, unless they accumulate secondary mutations that suppress the asporogenous phenotype. Such spores yield vegetative cells with significantly increased ability to form new spores compared to the asporogenous parental strain. For simplicity, we will refer to both asporogenous and oligosporogenous mutants as Spo^−^, and strains able to sporulate efficiently will be referred to as Spo^+^.

Researchers quickly devised simple, forward genetic screens to isolate Spo^−^ mutants in large numbers, and mapped the mutations using classical genetic methods such as transformation and transduction [27, 28]. Those studies were facilitated by the development of simple tests to distinguish Spo^+^ and Spo^−^ strains. Spo^−^ mutants form white colonies in certain rich media, whereas strains able to sporulate efficiently form brown colonies. The brown color is due to the accumulation of a pigment whose synthesis requires a component of the spore coat, CotA [29, 30]. Secondly, the heat resistance of the spores provides a definitive test to distinguish Spo^+^ and Spo^−^ strains [31]. Determining the linkage between mutations conferring the Spo^−^ phenotype and different markers in the chromosome led to the understanding that there are multiple sporulation (*spo*) loci, scattered around the chromosome [27, 28, 32–34].

The initial analysis of Spo^−^ mutants indicated that sporulation was blocked at different stages in different Spo^−^ mutants [31], suggesting that each sporulation stage was controlled by a dedicated set of genes. This was formally confirmed in 1966, when Ryter and collaborators performed a cytological characterization of *B. subtilis* strains carrying different *spo* mutations using electron microscopy, and classified them according to the stage at which sporulation was blocked [18]. This study led to the establishment of a specific nomenclature for the *spo* loci that was formalized in a classic review by Piggot and Coote in 1976 [19]. The *spo* loci were defined as those, that when mutated, impaired the ability of the cell to form spores, without having a noticeable effect on vegetative growth. The loci were classified into different categories (*spo0, spoII, spoIII, spoIV*, and so on), according to the stage at which sporulation was blocked in the mutants (**Fig. 2**). No distinction was made between stages 0 and I, and mutations that caused blockage in either stage were globally referred to as *spo0*. A capital letter was used to distinguish different loci, mutations in which produced blockages at the same stage. Thus, for example, *spoIIIA* and *spoIIIE* refer to two distinct loci that, when mutated, produce a blockage at stage III. In some cases, it was later discovered that a specific locus was actually an operon containing several genes. In those cases, the different genes of the operon were assigned a second capital letter (for example, the *spoIIIA* locus actually consists of an operon of at least eight genes, which are named in order from s*poIIIAA* to *spoIIIAH*).

**Figure 3:**
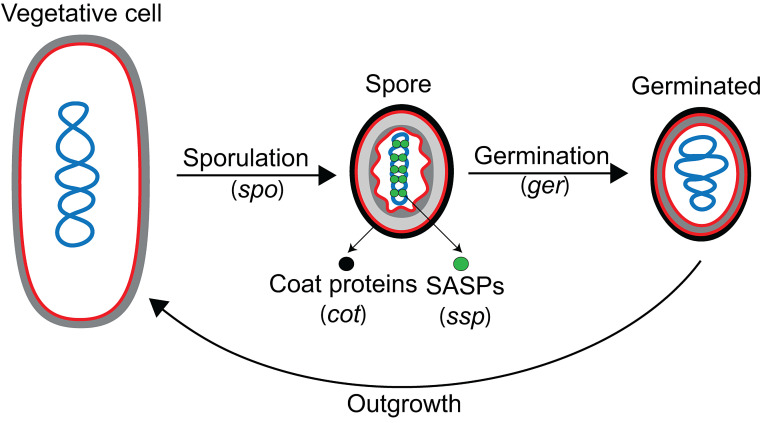
FIGURE 3: Developmental loci in *B. subtilis*. Diagram of the sporulation-germination cycle, with the different classes of developmental loci noted (in parentheses). The *spo* loci are required for spore formation, but not for vegetative growth. The *ger* loci are required for proper spore germination. After germination, metabolism is reactivated and the spore transforms into a growing vegetative cell in a process called outgrowth, which is thought to depend on the same pathways that control vegetative growth. The *cot* and *ssp* loci were identified after the isolation and characterization of coat and small acid-soluble proteins (SASPs), respectively, from spores. In addition to the these categories, there are hundreds of developmentally-regulated genes whose roles in sporulation and germination are not yet understood. Membranes, red; DNA, blue; cell wall, gray; spore cortex, light gray; spore coat, black; SASPs, green circles.

In addition to the *spo* genes, three more developmental gene categories were described: *ger, cot* and *ssp* (**Fig. 3**). Many *ger* genes were identified in elegant genetic screens for mutations that rendered spores unable to germinate properly [35–40]. These screens relied on an agar plate assay in which colonies formed by mutagenized cells were treated with heat or chloroform vapor to kill vegetative cells [35]. The remaining spores were then overlaid with agar containing two key components: (i) nutrients to induce germination, and (ii) 2,3,5-triphenyltetrazolium chloride (TZM), which turns red when reduced by metabolically-active cells. Spores are metabolically dormant, and are unable to reduce TZM to produce the red pigment, but upon germination become metabolically active and can reduce TZM to give the colonies a pink coloration. Colonies containing germination-deficient spores fail to reduce TZM, and therefore, do not produce the red pigment. While some of the *ger* genes identified in germination screens encode proteins involved exclusively in germination (for example, the genes encoding germinant receptors, such as those in the *gerA* and *gerB* operons), others encode proteins that are actually required for proper spore development, generating some overlap between *ger* and *spo* genes (for instance, *gerM* is required for proper sporulation, but it was first identified in a screen for germination mutants [41, 42]).

The two major remaining categories of developmental genes, *cot* (for coat) and *ssp* (for spore-specific protein), were identified by reverse genetics, after the isolation and biochemical characterization of coat proteins and small acid-soluble proteins (SASPs) [29, 43–47] from spores, respectively (**Fig. 3**). SASPs are abundant spore proteins that saturate and protect the spore chromosome [45]. Since individual SASPs and most coat proteins are dispensable for *B. subtilis* sporulation under standard laboratory conditions [29, 46, 48, 49], they were missed in the genetic screens that led to the identification of *spo* genes.

The discovery and characterization of developmental genes accelerated after the rise of DNA cloning and sequencing technology. Researchers were able to clone *spo* genes by selecting for chromosomal DNA fragments that complemented the respective *spo* mutations. From these clones, *lacZ* fusions were generated, and these in turn allowed for the study of the *spo* genes *in vivo*. New genetic tools, such as Tn*917*-based transposition mutagenesis [50–52] and integrational plasmid vectors [52–55], further facilitated the identification and manipulation of *spo* loci. During the late 1970's and 80's, many developmental loci were cloned and sequenced, their expression characterized, and the role of the proteins they encode started to be deciphered.

## CELL-SPECIFIC GENE EXPRESSION

**Figure 4:**
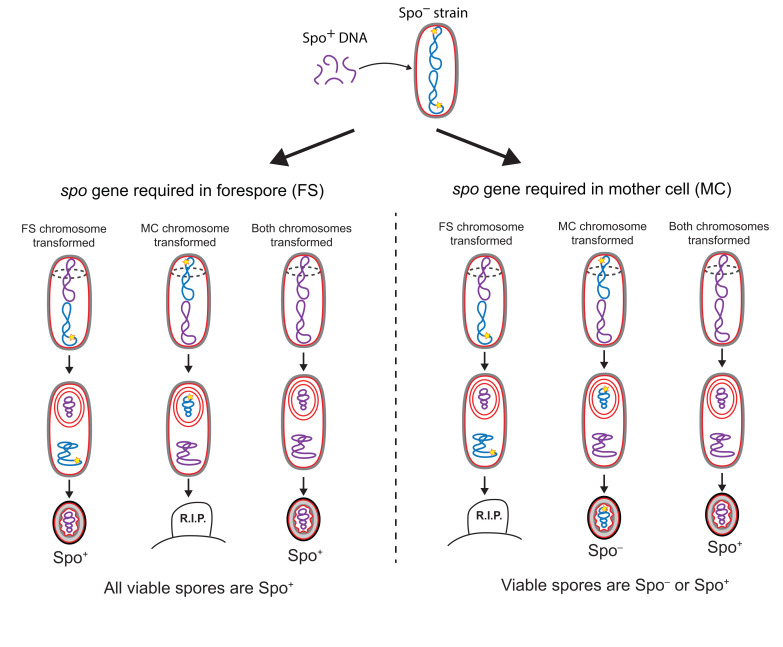
FIGURE 4: Genetic strategy to identify the cell in which a *spo* gene is required. A Spo^−^ strain with a mutation in a known *spo* gene (yellow star) is depicted in Stage I (see **Fig. 2**) with its two chromosomes, one that will be packed into the forespore (upper chromosome) and one that will remain in the mother cell and be destroyed when the mother cell lyses (lower chromosome). The strain is transformed with genomic DNA from a Spo^+^ strain at the onset of sporulation. Transformed chromosomes are drawn in purple, non-transformed chromosomes, in blue. Either or both chromosomes are capable of being transformed to *spo*^+^. If the *spo* gene is required in the forespore (**left panel**), transformation of the forespore chromosome to *spo*^+^ rescues the process and generates spores that can germinate and go on to sporulate again (left). But transformation of the mother-cell chromosome to *spo*^+^ with no accompanying transformation of the forespore chromosome leaves the forespore chromosome *spo*^−^ so the cells cannot complete the process, and no spores are produced (center). Hence only Spo^+^ spores are generated if the *spo* gene is required in the forespore. If the *spo* gene is required in the mother cell (**right panel**), transformation of the mother-cell chromosome to *spo*^+^ rescues the process (center and right), but only if the forespore chromosome is transformed as well will the spores that are produced be able to germinate into cells that can go on to sporulate again (right). Hence, both Spo^+^ and Spo^−^ spores can be generated if the *spo* gene is required in the mother cell.

Three main conclusions arose from the initial genetic analysis of sporulation: first, most developmental genes are not expressed in growing cells, and their expression is induced at different times during sporulation; second, there is a dependency hierarchy in developmental gene expression, such that the expression of genes that participate in later events in the sporulation pathway tends to depend on the expression of genes that participate in earlier events; third, the majority of the sporulation genes are expressed in only one of the two cells required to form a spore, either the mother cell or the forespore. This last conclusion was achieved by using fractionation techniques to separate forespore and mother-cell protoplasms [56–58], and by the implementation of genetic strategies to determine the cell in which different *spo* genes were required [59, 60]. In one such genetic strategy, developed by Lencastre and Piggot [59] and illustrated in **Fig. 4**, Spo^−^ mutants were transformed with genomic DNA from the wild-type (Spo^+^) strain at the start of sporulation, thereby rendering the Spo^−^ mutants capable of forming heat-resistant spores. The parental Spo^−^ mutants fell into two categories, depending on the ability of the resultant spores to produce additional spores following germination: mutants in the first category produced spores that were homogeneously Spo^+^; mutants in the second category produced a heterogeneous population of Spo^+^ and Spo^−^ spores. The *spo* genes mutated in the first category of parental strains were inferred to be required in the forespore, and the ones mutated in the second were inferred to be required in the mother cell. The logic underlying this interpretation is as follows (**Fig. 4**): of the two cells required to form a spore, only the forespore retains its chromosome in the mature spore, since the mother cell lyses upon sporulation completion (**Fig. 2**). Thus, *spo* genes required in the forespore must be complemented in the forespore chromosome and the wild-type version of the gene will therefore always present in mature spores, and these will give rise to a homogenous population of Spo^+^ transformant spores. But if a *spo* gene is required in the mother cell and not in the forespore, then sporulating cells in which only the mother-cell chromosome is transformed with the wild-type allele will be able to form mature spores, but the non-transformed forespore chromosome would still contain the mutated version of the *spo* gene, and the spores would remain Spo^−^.

Results from these biochemical and genetic analyses underscored that different genetic programs were activated in the two cells, at different times during sporulation. From here, the focus moved on to deciphering the genetic regulation of sporulation.

In 1969, it was discovered that bacterial RNA polymerases contain a factor, called Sigma (σ), that is necessary for promoter recognition and transcription initiation [61, 62]. This finding led researchers to hypothesize the existence of alternative σ factors, which could direct RNA polymerase to specific subsets of promoters. The existence of such alternative σ factors was first demonstrated in *B. subtilis* phage SP01 [63], which encodes σ factors that drive expression from middle and late phage promoters. The first bacterial alternative σ factor to be discovered was α^37^ [64], a general-stress response σ factor of *B. subtilis.* Initially, the name of the different σ factors was based on their molecular weight, in kDa. Thus, α^37^ was ~37 kDa, as determined by the apparent size of the purified protein in an acrylamide gel. However, the discovery of alternative σ factors with similar sizes or that migrated aberrantly in acrylamide gels accelerated over the following years, which led researchers in the *B. subtilis* field to replace the molecular weight designation with a letter reflecting order of discovery [65, 66]. Thus, σ^37^ was renamed σ^B^, the name by which it is commonly known today, and the genes encoding the σ factors were named *sig* followed by their corresponding letter.

**Figure 5:**
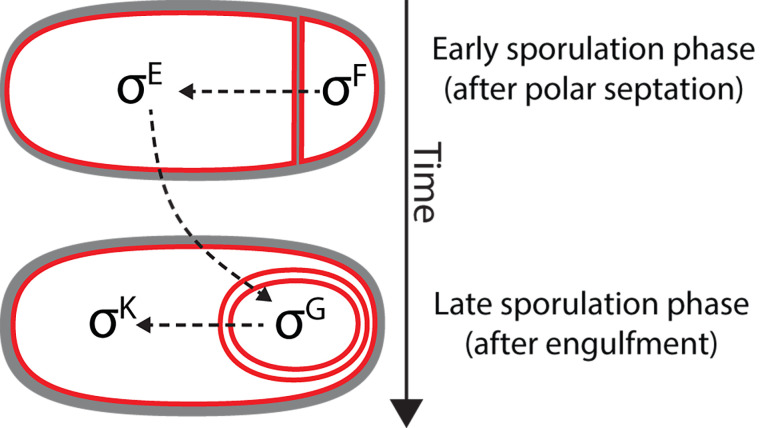
FIGURE 5: Cascade of sporulation-specific σ factors in *B. subtilis*. Immediately after polar septation, σ^F^ is activated in the forespore. σ^F^ activation triggers an intercompartmental signaling cascade that leads to the activation of σ^E^ in the mother cell. Together σ^F^ and σ^E^ control gene expression during engulfment. Roughly coincident with the completion of engulfment, σ^F^ is replaced by σ^G^ in the forespore, which leads to the subsequent activation of σ^K^ in the mother cell. σ^G^ and σ^K^ control gene expression in the forespore and the mother cell, respectively, after engulfment.

Shortly after the discovery of σ^B^, it was shown that the sporulation regulatory program also relies on alternative σ factors that are activated in the mother cell or in the forespore at different developmental stages. The first sporulation-specific σ factor to be discovered was σ^E^ (originally referred to as σ^29^) [67]. σ^E^ was discovered using predominantly biochemical and molecular biology techniques [67]. The protein was isolated from RNA polymerase purified from sporulating cultures, and was shown to change the promoter specificity of RNA polymerase *in vitro*. Not surprisingly, it was subsequently shown that σ^E^ was encoded by a previously identified *spo* locus, *spoIIGB* (later renamed as *sigE*) [68, 69]. After σ^E^, three additional sporulation σ factors were identified by means of both sequence similarity with previously identified σ factors, and *in vitro* transcription assays of sporulation genes: σ^F^ [70, 71], σ^G^ [72–74] and σ^K^ [75]. In *B. subtilis*, the activation of the different sporulation σ factors follows a hierarchical order in which different σ factors are sequentially activated in alternating cellular compartments, and the activation of a later factor depends on the activation of the previous one (**Fig. 5**): first, σ^F^ becomes active in the forespore shortly after polar septation, followed by σ^E^ in the mother cell. Roughly coincident with engulfment completion, σ^G^ is activated in the forespore and, finally, σ^K^ is activated in the mother cell. The variety of mechanisms involved in the sequential activation of these σ factors includes phosphorelays, proteolytic processing, and even a gene splicing event [76–79].

After the discovery of the sporulation-specific σ factors, many of the developmental genes previously identified were assigned to different regulons, depending on the σ factor controlling their expression. Microarray technology allowed researchers to discover additional genes under the control of sporulation σ factors [80, 81]. Together the cell-specific σ factors control the expression of more than 500 different genes [82, 83], the majority of which are not expressed during vegetative growth. Over the course of the past few decades, the study of sporulation has focused on deciphering the function of genes that are under the control of sporulation-specific σ factors. But the functions of nearly 1/3 of these genes remain a mystery, and for the majority of sporulation genes with assigned function, we have only a superficial knowledge of how specific gene products contribute to spore formation. We envision that the study of developmentally-regulated genes will continue to be a fruitful area of research for years to come.

## CELL-SPECIFIC GENE EXPRESSION AS A GENETIC TOOL

**Figure 6:**
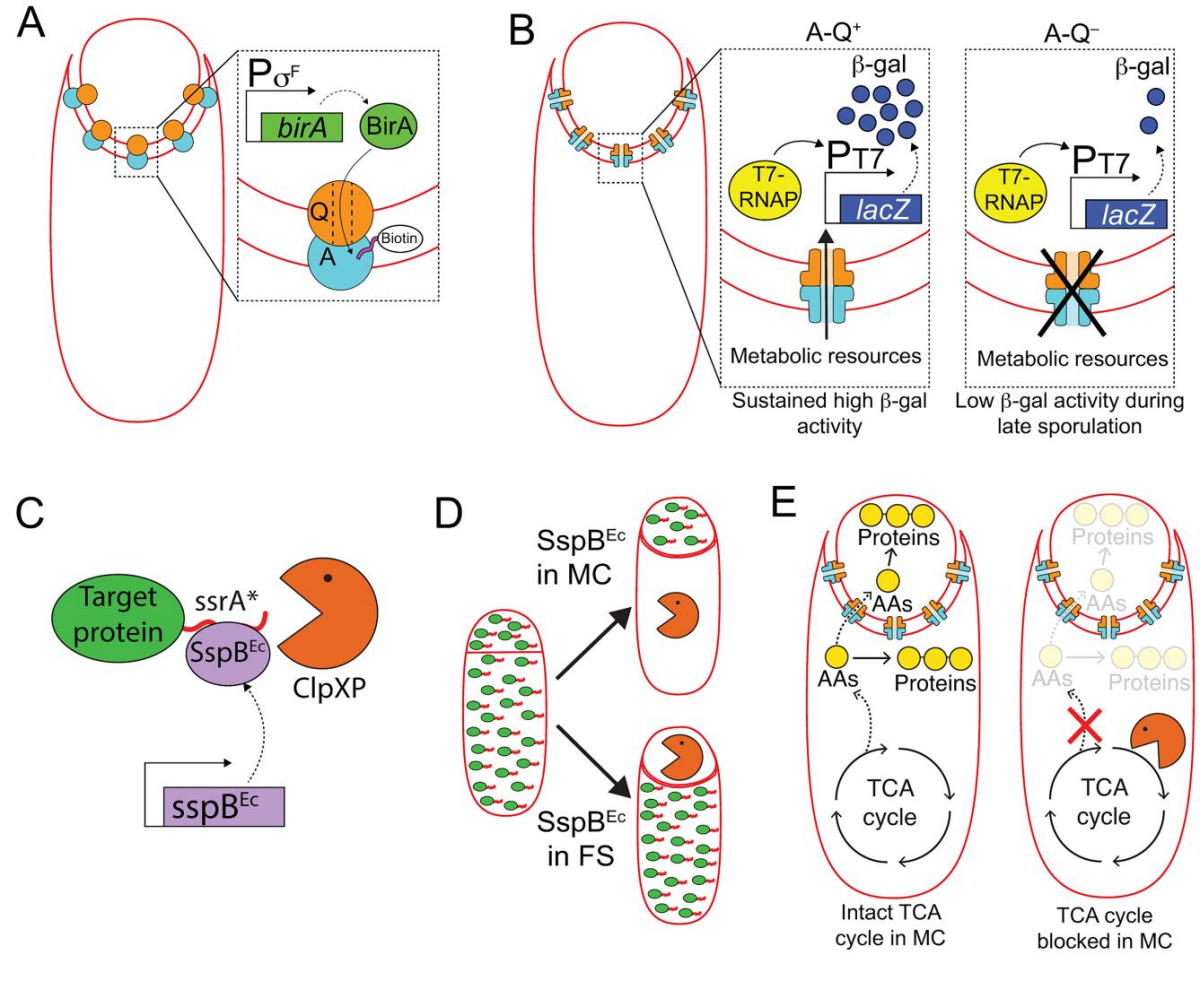
FIGURE 6: Harnessing cell-specific gene expression to study the function of A-Q complexes. **(A)** The forespore protein SpoIIQ (Q, orange circle) and the mother-cell proteins encoded in the *spoIIIA* operon (A, light blue circle), form trans-envelope complexes that bridge the forespore and mother-cell membranes during engulfment. Zoomed in panel: Biotin ligase (BirA, green) produced in the forespore is able to biotinylate a biotin acceptor peptide (pink) fused to the extracellular domain of the A protein, SpoIIIAH [111], indicating that A-Q complexes are channels (see panel B), and that the channel pore is open on the forespore side and large enough for BirA to reach the extracellular domain of SpoIIIAH. **(B)** The activity of a heterologous RNA polymerase (T7 RNAP, yellow) produced in the forespore under σ^F^ control is monitored by the accumulation of β-galactosidase (β-gal, dark blue circles) produced through the expression of a *lacZ* gene under the control of a T7 RNAP-dependent promoter. In the presence of A-Q channels (A-Q^+^, left zoomed in panel), sustained β-galactosidase activity is detected throughout sporulation. In the absence of A-Q channels (A-Q^−^, right zoomed in panel), β-galactosidase activity drops at later sporulation stages. Camp and Losick [112] proposed that A-Q channels constitute feeding tubes through which the mother cell transfers metabolic resources to the forespore to maintain biosynthetic activities at late sporulation stages. **(C)** The ssrA*/SspB^Ec^ inducible protein degradation system [114]. SspB^Ec^ (purple) binds to ssrA* (red) fused to the C-terminus of target proteins (green), and delivers the target proteins to the endogenous *B. subtilis* protease ClpXP (orange pacman) for degradation. **(D)** Cell-specific degradation of target proteins during sporulation is achieved by expressing *sspB*^*Ec*^ from mother cell- or forespore-specific promoters [113]. Target proteins represented by green ovals tagged with ssrA (red line); degradation represented by orange pacman. **(E)** Left cell: Mother-cell TCA cycle provides metabolic precursors, such as amino acids (AAs, yellow circles), to support protein synthesis in both the mother cell and the forespore [115]. Mother-cell metabolic precursors could be transported to the forespore via A-Q channels, in keeping with the feeding tube model. Right cell: Degradation of TCA cycle enzymes in the mother cell blocks protein synthesis in both the mother cell and the forespore [115].

Beyond its central importance to the developmental program of sporulation, cell-specific gene expression has come to provide a powerful genetic tool for the study of different aspects of spore formation. After the discovery of the sporulation-specific σ factors, many researchers carefully characterized promoters regulated by the different σ factors (i.e. [84–92]), enabling these promoters to be co-opted for expression of heterologous genes in either the mother cell or the forespore, at different developmental stages. Cell-specific gene expression has been used to study many of the fundamental features of spore formation such as the temporal compartmentalization of the different sporulation σ factors [93], the organization of the chromosomes after sporulation initiation [94], and the transport of the forespore chromosome from the mother cell to the forespore [95–101], among other things. In order to illustrate the power of this approach, we describe three experiments that have helped to disentangle, at least partially, the function of the sporulation loci *spoIIIA* and *spoIIQ* (**Fig. 6**):

Mother-cell proteins encoded in the *spoIIIA* operon (which contains eight genes, *spoIIIAA* to *spoIIIAH*) together with the forespore protein SpoIIQ form a trans-envelope multimeric complex (henceforth A-Q complexes) that spans the mother-cell and forespore membranes (**Fig. 6A**) [102, 103]. Mutants lacking *spoIIIA* or *spoIIQ* are asporogenous, as they fail to activate the late forespore σ factor, σ^G^ [104, 105]. Some of the SpoIIIA proteins show sequence and structural homology to components of various secretion systems from Gram-negative bacteria [103, 106–108], and one of them, SpoIIIAG, has been shown to form multimeric rings *in vitro* [109, 110], leading to the proposal that A-Q complexes constitute proteinaceous channels that connect the mother cell and the forespore, through which molecules required for σ^G^ activation might be transferred between the two cells. Meisner and collaborators developed a compartmentalized biotinylation assay to test this idea (**Fig. 6A**) [111]. The authors used mother cell- and forespore-specific promoters to express the gene encoding *E. coli* biotin ligase (*birA*) in either cell, and fused biotin acceptor peptides to the extracellular domains of A and Q proteins. They found that BirA produced in the forespore could biotinylate the extracellular domain of SpoIIIAH, which supported the idea that A-Q complexes are channels that are open at least on the forespore side.

In a separate study, Camp and Losick took advantage of cell-specific gene expression to study whether A-Q channels were specifically required for σ^G^ activation, or if they also played a role in the expression of σ^G^-independent genes (**Fig. 6B**) [112]. They monitored the activity of the heterologous RNA polymerase, T7 RNAP, when produced in the forespore from a σ^F^-dependent promoter by placing a *lacZ* gene under the control of a T7 RNAP-dependent promoter. In the presence of A-Q, β-galactosidase activity could be detected at the onset of sporulation, and it increased gradually over time until it reached a plateau that was maintained throughout spore formation. In mutants lacking functional A-Q complexes, β-galactosidase activity was also detected at the onset of sporulation, but dropped off later in sporulation, at roughly the time when σ^G^ would be normally activated. These observations indicated that A-Q plays a general role in regulating forespore gene expression at late sporulation stages, rather than affecting σ^G^ activity specifically. The authors proposed that A-Q channels constitute a feeding tube through which the mother cell provides metabolic resources to the forespore in order to maintain forespore biosynthetic activities at late sporulation stages [112].

We have recently accumulated more evidence that the mother cell nurtures the forespore, by implementing a cell-specific protein degradation system that also relies on cell-specific gene expression (**Fig. 6C** and **D**) [113, 114]. The system uses modified ssrA* tags from *E. coli* fused to the C-terminus of target proteins [114], and the cognate *E. coli* SspB adaptor protein produced under the control of mother-cell or forespore-specific promoters [113] (note that *E. coli* SspB is not related to the sporulation *ssp* genes described previously in this manuscript; to avoid confusion, *E. coli* SspB will be referred to as SspB^Ec^). When SspB^Ec^ is produced, it binds to ssrA* and delivers the target protein to the endogenous *B. subtilis* protease ClpXP for degradation (**Fig. 6C** and **D**) [114]. We are using this platform to systematically assess the requirement of different metabolic pathways in the mother cell and in the forespore for sporulation to progress normally. Our results indicate that the tricarboxylic acid (TCA) cycle is required in the mother cell, but is largely dispensable in the forespore (**Fig. 6E**) [115]. We have also observed that protein synthesis in the forespore requires TCA cycle activity in the mother cell, suggesting that the mother cell produces TCA cycle-derived metabolic precursors, such as amino acids, for protein synthesis in both the mother cell and the forespore (**Fig. 6E**) [115]. These findings indicate that mother-cell metabolic precursors are transported to the forespore to support protein synthesis, in line with the feeding tube model proposed by Camp and Losick [112].

Overall, cell-specific gene expression has been a fantastic addition to the list of genetic tools available in *B. subtilis*. We are looking forward to seeing new creative approaches using those tools to explore the inner workings of sporulating bacteria.

## THE GENETIC BASIS OF CELLULAR DYNAMICS

The study of sporulation has helped to overturn the traditional dogma that bacterial cells lack a defined internal organization owing to the absence of membrane-bound organelles. Over the last 25 years, we have obtained precise descriptions of how proteins are redistributed during sporulation to mediate dynamic cellular processes. Advances in our understanding of sporulation during this period have been driven by the development of a wealth of new imaging technologies, particularly in fluorescence microscopy, which have allowed the field of bacterial cell biology to emerge. We illustrate the rapid progress made in this field by focusing on a single protein, SpoIIIE.

**Figure 7:**
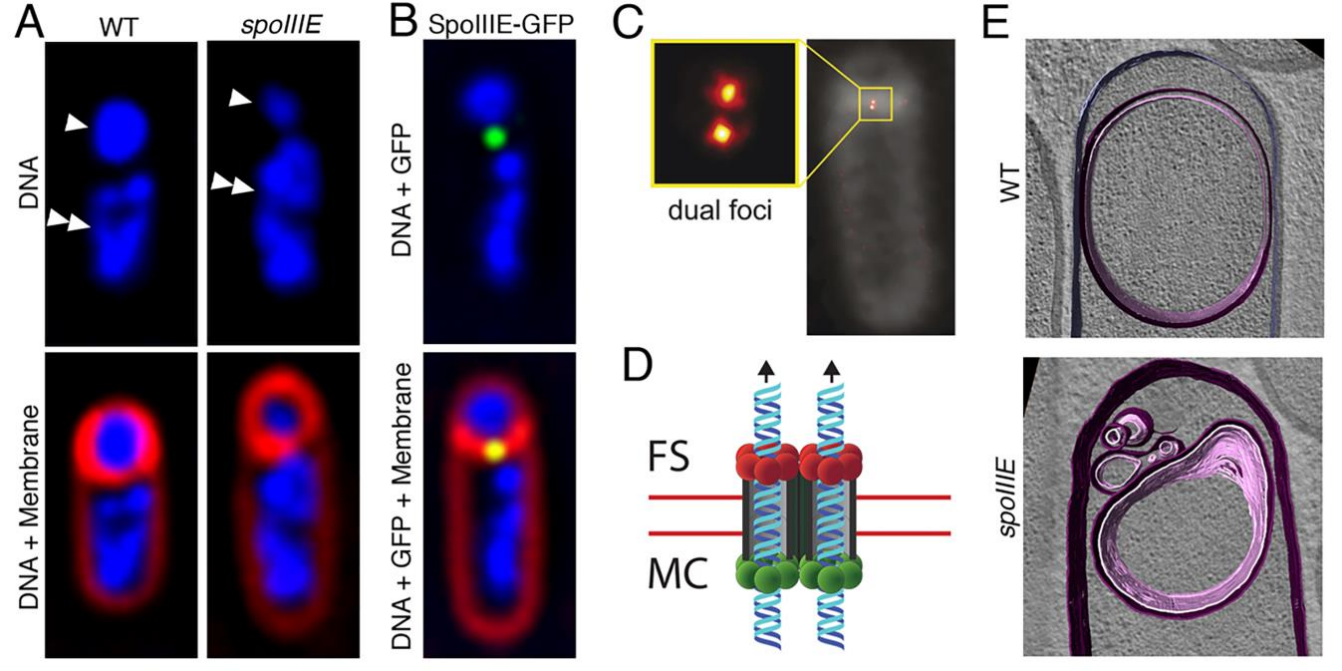
FIGURE 7: Progress in our understanding of SpoIIIE-mediated chromosome translocation. **(A)** Fluorescence microscopy images of a wild-type (WT, left) and of a *spoIIIE* mutant (*spoIIIE*, right) strain of *B. subtilis*. The upper panels show the DNA stained with DAPI (blue), and the lower panels the overlay of DAPI-stained DNA and the membranes stained with FM 4-64 (red). In the upper panel, forespores are indicated by single arrowheads, and mother cells by double arrowheads. Wild-type forespores contain a complete chromosome. However, forespores of *spoIIIE* mutant strains contain only ~30% of a chromosome, and the rest remains trapped in the mother cell. **(B)** Fluorescence microscopy image of a sporulating cell producing a SpoIIIE-GFP fusion during chromosome translocation. The upper panel shows the DNA stained with DAPI (blue) and the GFP signal, and the lower panel shows in addition the membranes stained with FM 4-64 (red). SpoIIIE forms a focus (green) at septal midpoint, where the chromosome is trapped. **(C)** SpoIIIE visualized by super-resolution microscopy (PALM) in living cells with thicker polar septa. SpoIIIE forms two foci (dual foci), which are separated by a distance equivalent to the septal thickness, indicating that one cluster is present at one side of the septum and the other at the opposite side. Reproduced from [101]. **(D)** Model for the organization and function of the SpoIIIE translocation complex at the septal midpoint. SpoIIIE forms two side-by-side channels spanning both septal membranes (red lines), thereby allowing the simultaneous transport of both arms of the chromosome from the mother cell (MC) to the forespore (FS). Translocation is powered by SpoIIIE motor domains at the mother-cell side of the septum (green circles), which are activated to export the chromosome to the forespore. Forespore motor domains (red circles) remain inactive. **(E)** Cryo-electron microscopy of a wild-type sporulating cell (top), and of a *spoIIIE* mutant (bottom). Membranes are annotated as follows: forespore membrane, pink; mother-cell membrane, purple. Chromosome translocation is required to maintain the shape of the forespore. In the absence of chromosome translocation, the forespore appears deflated. Reproduced with permission from [100].

Since the mid 20^th^ century, it was recognized that the two chromosomes resulting from a replication event prior to sporulation initiation are segregated during sporulation, such that one remains in the mother cell and the other is packed into the small forespore [12]. In 1994, Wu and Errington [116] reported that mutations in *spoIIIE*, one of the *spo* loci that was identified during the genetic analysis of sporulation mutants, prevented complete segregation of the forespore chromosome, such that only ~30% of the chromosome was present in the forespore with the rest still in the mother cell (**Fig. 7A**). They inferred that the polar septum trapped the forespore chromosome, and that SpoIIIE mediated the translocation of the chromosome across the septum into the forespore.

Further studies showed that SpoIIIE is a membrane-anchored FtsK-like protein that has a large C-terminal cytoplasmic motor domain [117] with DNA-dependent ATPase activity, that is capable of tracking along DNA in the presence of ATP [118]. More recently, *in vitro* studies with purified SpoIIIE motor domains have shown that they assemble into hexameric rings [119, 120], with an inner diameter large enough to accommodate a double-stranded DNA molecule [119]. Single molecule experiments using optical tweezers have allowed the characterization of the inter-subunit coordination [121] and of the mechanochemistry [122] of the hexameric rings as they move along DNA molecules, providing exquisite details of the mechanism of chromosome translocation at the molecular level.

SpoIIIE assembles into a translocation complex at the septal midpoint during the early stages of sporulation (**Fig. 7B**). This was shown first in fixed cells using immunofluorescence microscopy [117] and later in living cells with a SpoIIIE-GFP fusion and fluorescent membrane dyes that are compatible with live cell imaging [123] (**Fig. 7B**). More recently, studies of the assembly of the SpoIIIE translocation complex with super-resolution optical microscopy techniques [101, 124, 125] have revealed that SpoIIIE oligomers localize to the leading edge of the polar septum as it forms, and ultimately assemble a stable translocation complex at the septal midpoint. In mutants that produce thicker than normal polar septa, super-resolution microscopy has made it possible to resolve two different foci within the SpoIIIE complex (**Fig. 7C**), suggesting that the complex consists of two subcomplexes of opposite polarity, one anchored to the mother cell and the other to the forespore septal membrane. Each subcomplex contains enough SpoIIIE monomers to assemble at least two hexameric rings [101], which would allow the two arms of the chromosome to be translocated in parallel from the mother cell to the forespore (**Fig. 7D**) [98].

Assembly of the SpoIIIE translocation complex requires that the DNA be trapped at the septum [126], and the complex is in turn required to mediate septal membrane fission while the chromosome is translocated [124]. This has led to the proposal that the mother-cell and forespore SpoIIIE subcomplexes pair to form a continuous channel spanning both septal membranes (**Fig. 7D**). In support of this idea, it has been shown that both forespore and mother-cell subcomplexes are required to keep the septal membranes separated during chromosome translocation [101]. However, the mother-cell subcomplex by itself is sufficient to mediate the transport of the chromosome into the forespore [95, 101]. If the SpoIIIE mother-cell subcomplex is absent, the forespore subcomplex can transport the chromosome out of the forespore and into the mother cell [95, 101], indicating that the SpoIIIE translocation complex can, in principle, function as a bidirectional motor. During sporulation, however, the SpoIIIE translocation complex always transports the chromosome from the mother cell to the forespore (**Fig. 7D**). The mechanism that determines translocation directionality has been at least partially elucidated by means of a combination of *in vivo, in vitro* and *in silico* approaches. The motor domain of SpoIIIE is able to recognize specific, highly-skewed octameric sequences in the DNA called SpoIIIE Recognition Sequences (SRSs), which are present in each arm of the chromosome but on opposite strands [97]. Since the *B. subtilis* chromosome is circular and the two chromosome arms are trapped at the septum, the opposite orientation of SRSs on each arm might serve as a cue to determine translocation directionality. In fact, it has been shown that the interaction of SpoIIIE with the SRS in the orientation preferentially encountered by SpoIIIE as each chromosome arm is directed into the forespore stimulates SpoIIIE ATPase activity [120, 127, 128]. This might account for the direction of translocation and the simultaneous transport of both chromosome arms to the forespore.

In addition to guaranteeing that the forespore receives a full complement of genetic material, SpoIIIE-mediated chromosome translocation also contributes to the generation and maintenance of forespore shape during engulfment [100]. Packaging an entire chromosome into the small forespore compartment leads to a high turgor pressure, which inflates the forespore like a balloon and distends the forespore membranes (**Fig. 7E**).

While *spoIIIE* is among the best characterized *spo* loci, the field has made significant progress in the understanding of a myriad of dynamic processes associated with sporulation, such as the formation of the polar septum [129–131], the migration of the mother-cell membrane around the forespore during engulfment [132–139], the synthesis of the peptidoglycan cortex [140], and the assembly of the proteinaceous coat [20, 141], to name a few.

## HISTORICAL CONTINGENCIES

Sporulation in *B. subtilis* is one of the most well-understood developmental processes, but there are still gaps in our understanding, which are due in part to the historical contingencies of sporulation research. Much of the sporulation research performed so far has focused on understanding the role of different developmental genes in spore formation. The definition of *spo* genes—those that, when mutated, impair the ability to form spores without affecting vegetative growth—has likely biased our understanding of sporulation, as it necessarily excluded all the essential genes and non-essential genes that are also required for optimal vegetative growth. It has therefore remained unclear whether essential housekeeping proteins involved in central metabolism, redox reactions, protein folding, and translational regulation play a significant role in assembling the spore, and if so, if they are required in a specific cell or during a specific stage of sporulation. Recent high-throughput studies have identified vegetative proteins that likely play a role in sporulation [142, 143]. Genetic tools to interfere with the function of specific proteins in a precise, cell- and developmental stage-specific manner during spore formation have also recently been developed (**Fig. 6C** and **D**) [113], and these present a promising new approach for understanding the role played by these and other vegetative proteins during endospore formation. Although many facets of sporulation remain unclear, new technologies and innovative approaches will continue to advance our knowledge of this remarkable process.

A more general aspect of endospore formation that remains poorly studied is how the process functions in different species. Endospore formation occurs in a broad range of bacterial species that belong to an ancient and exceptionally diverse bacterial phylum, the Firmicutes [144]. However, most mechanistic studies on sporulation over the previous 50 years have been done in *B. subtilis*, as this organism is particularly amenable to genetic studies. Placing the focus on a single species has provided us with extraordinarily detailed descriptions of sporulation but, at the same time, has prevented us from fully appreciating the diversity of endospore formation. In recent years, *Clostridioides difficile* —formerly known as *Clostridium difficile*—has become a model for endospore formation in anaerobic bacteria. Studies in this organism reveal that, although the general scheme of endospore formation is similar to that of *B. subtilis*, there are mechanistic differences that affect every sporulation stage, from initiation to germination [145–151]. In addition, while *B. subtilis* and *C. difficile* produce one spore per sporangium, there are some species that can produce two [152, 153], and others that can produce multiple spores [154–156], opening the intriguing possibility that the sporulation pathway was co-opted as a reproductive strategy in some species, or that sporulation evolved from what was originally a reproductive strategy [157–160]. Clearly, this is a rich and interesting area of future research that will require the development of new culturing techniques and genetic tools to manipulate non-model bacteria. Understanding the diversity of endospore formation may allow us to address key questions regarding the ecological roles and the evolutionary origin of this fascinating developmental process.

## Abbreviations:

SASP: – small acid-soluble protein,
Spo^−^: – asporogenous and oligosporogenous mutants,
Spo^+^: – sporulating strains,
SRS: – SpoIIIE Recognition Sequence,
TCA: – tricarboxylic acid,
TZM: – 2,3,5-triphenyltetrazolium chloride.
